# Supplementary material for: SARS-CoV-2 Spike Glycoprotein and ACE2 Interaction Reveals Modulation of Viral Entry in Wild and Domestic Animals
Source: Front Med (Lausanne). 2022 Mar 11;8:775572. doi: 10.3389/fmed.2021.775572 (PMC8962831; doi:10.3389/fmed.2021.775572)
Supplement: Data Sheet 1 — Nucleotide sequence alignment of the coding sequence (CDS) region of angiotensin-converting enzyme 2 (ACE2). The shaded regions show the spike interacting domains. [file Data_Sheet_1.PDF]

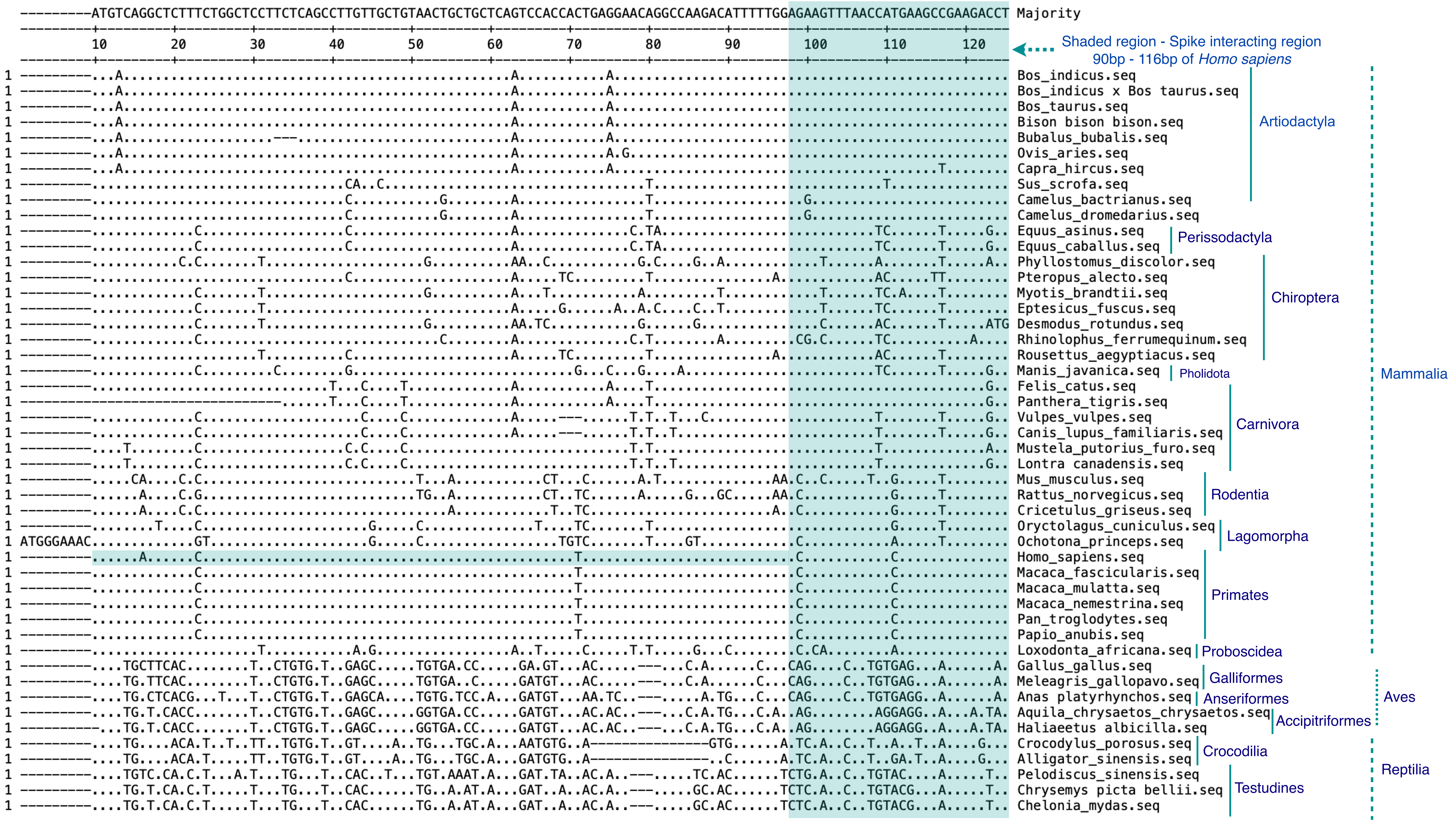

ATGTCAGGCTCTTTCTGGCTCCTTCTCAGCCTTGTTGCTGTAACTGCTGCTCAGTCCACCACTGAGGAACAGGCCAAGACATTTTTGGAGAAGTTTAACCATGAAGCCGAAGACCT Majority
10 20 30 40 50 60 70 80 90 100 110 120
Shaded region - Spike interacting region
90bp - 116bp of Homo sapiens
Bos_indicus.seq
Bos_indicus x Bos taurus.seq
Bos_taurus.seq
Bison bison bison.seq
Bubalus_bubalis.seq
Ovis_aries.seq
Capra_hircus.seq
Sus_scrofa.seq
Camelus_bactrianus.seq
Camelus_dromedarius.seq
Artiodactyla
Equus_asinus.seq
Equus_caballus.seq
Perissodactyla
Phyllostomus_discolor.seq
Pteropus_alecto.seq
Myotis_brandtii.seq
Eptesicus_fuscus.seq
Desmodus_rotundus.seq
Rhinolophus_ferrumequinum.seq
Rousettus_aegyptiacus.seq
Chiroptera
Manis_javanica.seq
Pholidota
Felis_catus.seq
Panthera_tigris.seq
Vulpes_vulpes.seq
Canis_lupus_familiaris.seq
Mustela_putorius_furo.seq
Lontra canadensis.seq
Carnivora
Mus_musculus.seq
Rattus_norvegicus.seq
Cricetulus_griseus.seq
Rodentia
Oryctolagus_cuniculus.seq
Ochotona_princeps.seq
Lagomorpha
Homo_sapiens.seq
Macaca_fascicularis.seq
Macaca_mulatta.seq
Macaca_nemestrina.seq
Pan_troglodytes.seq
Papio_anubis.seq
Primates
Loxodonta_africana.seq
Proboscidea
Mammalia
Gallus_gallus.seq
Meleagris_gallopavo.seq
Galliformes
Anas platyrhynchos.seq
Anseriformes
Aquila_chrysaetos_chrysaetos.seq
Haliaeetus albicilla.seq
Accipitriformes
Aves
Crocodylus_porosus.seq
Alligator_sinensis.seq
Crocodilia
Pelodiscus_sinensis.seq
Chrysemys picta bellii.seq
Chelonia_mydas.seq
Testudines
Reptilia

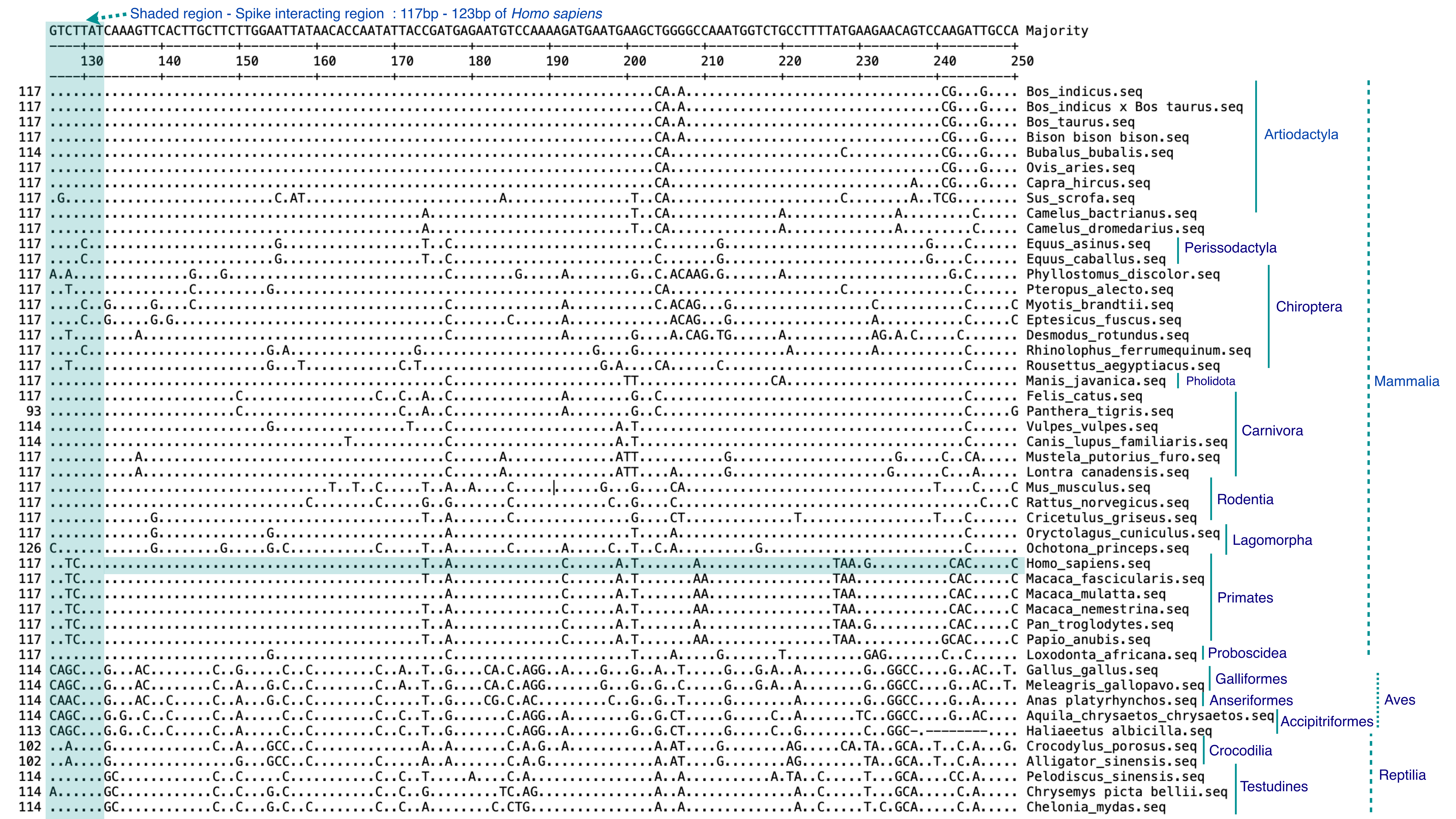

Shaded region - Spike interacting region : 117bp - 123bp of *Homo sapiens*
GTCTTATCAAAGTTCACTTGCTTCTTGGAATTATAACACCAATATTACCGATGAGAATGTCCAAAAGATGAATGAAGCTGGGGCCAAATGGTCTGCCTTTTATGAAGAACAGTCCAAGATTGCCA Majority
130 140 150 160 170 180 190 200 210 220 230 240 250
Bos_indicus.seq
Bos_indicus x Bos taurus.seq
Bos_taurus.seq
Bison bison bison.seq
Bubalus_bubalis.seq
Ovis_aries.seq
Capra_hircus.seq
Sus_scrofa.seq
Camelus_bactrianus.seq
Camelus_dromedarius.seq
Artiodactyla
Equus_asinus.seq
Equus_caballus.seq
Perissodactyla
Phyllostomus_discolor.seq
Pteropus_alecto.seq
Myotis_brandtii.seq
Eptesicus_fuscus.seq
Desmodus_rotundus.seq
Rhinolophus_ferrumequinum.seq
Rousettus_aegyptiacus.seq
Chiroptera
Manis_javanica.seq
Pholidota
Felis_catus.seq
Panthera_tigris.seq
Vulpes_vulpes.seq
Canis_lupus_familiaris.seq
Mustela_putorius_furo.seq
Lontra canadensis.seq
Carnivora
Mus_musculus.seq
Rattus_norvegicus.seq
Cricetulus_griseus.seq
Rodentia
Oryctolagus_cuniculus.seq
Ochotona_princeps.seq
Lagomorpha
Homo_sapiens.seq
Macaca_fascicularis.seq
Macaca_mulatta.seq
Macaca_nemestrina.seq
Pan_troglodytes.seq
Papio_anubis.seq
Primates
Loxodonta_africana.seq
Proboscidea
Mammalia
Gallus_gallus.seq
Meleagris_gallopavo.seq
Galliformes
Anas platyrhynchos.seq
Anseriformes
Aquila_chrysaetos_chrysaetos.seq
Haliaeetus albicilla.seq
Accipitriformes
Aves
Crocodylus_porosus.seq
Alligator_sinensis.seq
Crocodilia
Pelodiscus_sinensis.seq
Chrysemys picta bellii.seq
Chelonia_mydas.seq
Testudines
Reptilia

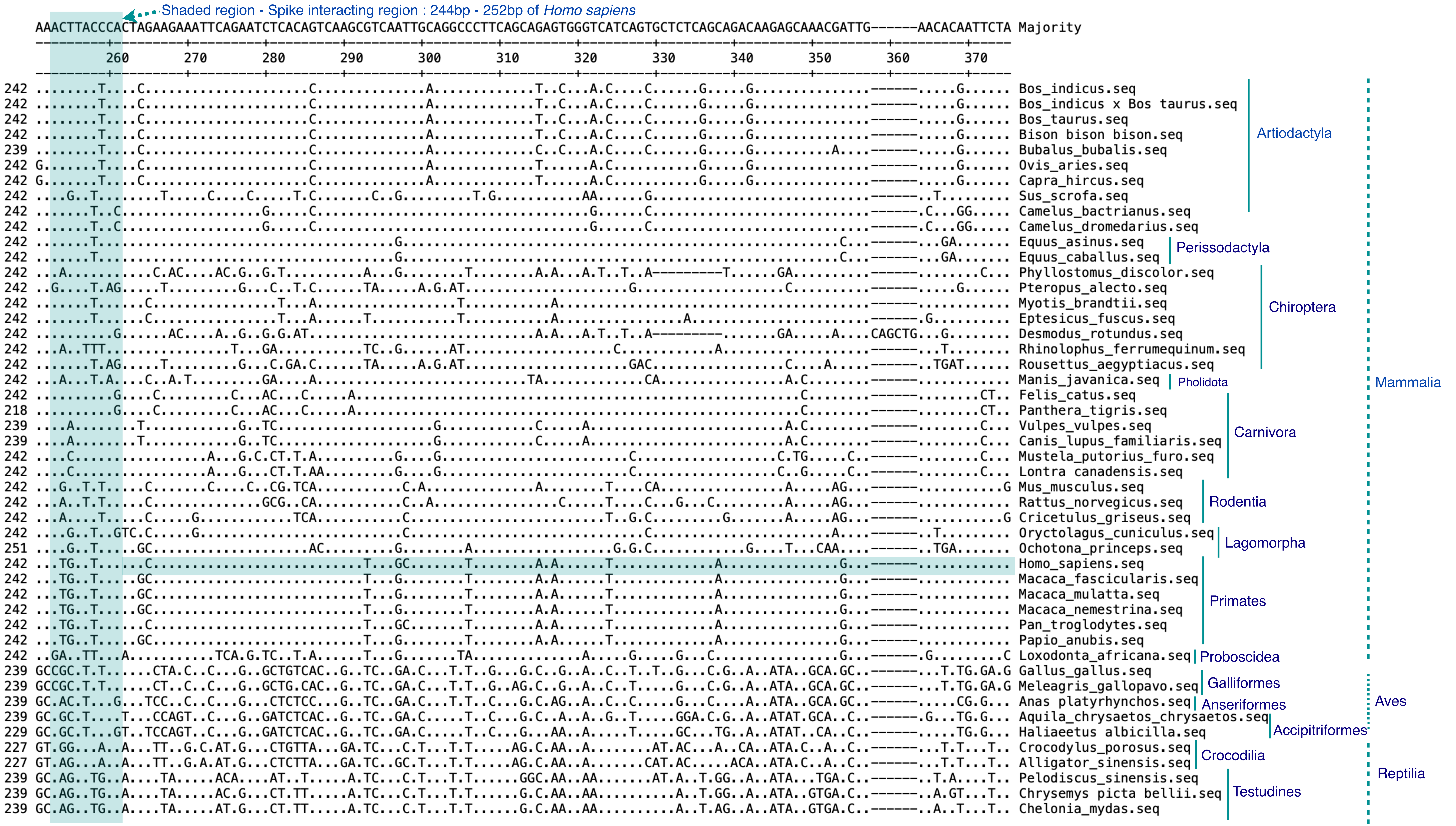

Shaded region - Spike interacting region : 244bp - 252bp of *Homo sapiens*
Majority
260
270
280
290
300
310
320
330
340
350
360
370
Bos_indicus.seq
Bos_indicus x Bos taurus.seq
Bos_taurus.seq
Bison bison bison.seq
Bubalus_bubalis.seq
Ovis_aries.seq
Capra_hircus.seq
Sus_scrofa.seq
Camelus_bactrianus.seq
Camelus_dromedarius.seq
Equus_asinus.seq
Equus_caballus.seq
Phyllostomus_discolor.seq
Pteropus_alecto.seq
Myotis_brandtii.seq
Eptesicus_fuscus.seq
Desmodus_rotundus.seq
Rhinolophus_ferrumequinum.seq
Rousettus_aegyptiacus.seq
Manis_javanica.seq
Felis_catus.seq
Panthera_tigris.seq
Vulpes_vulpes.seq
Canis_lupus_familiaris.seq
Mustela_putorius_furo.seq
Lontra canadensis.seq
Mus_musculus.seq
Rattus_norvegicus.seq
Cricetulus_griseus.seq
Oryctolagus_cuniculus.seq
Ochotona_princeps.seq
Homo_sapiens.seq
Macaca_fascicularis.seq
Macaca_mulatta.seq
Macaca_nemestrina.seq
Pan_troglodytes.seq
Papio_anubis.seq
Loxodonta_africana.seq
Gallus_gallus.seq
Meleagris_gallopavo.seq
Anas platyrhynchos.seq
Aquila_chrysaetos_chrysaetos.seq
Haliaeetus albicilla.seq
Crocodylus_porosus.seq
Alligator_sinensis.seq
Pelodiscus_sinensis.seq
Chrysemys picta bellii.seq
Chelonia_mydas.seq
Artiodactyla
Perissodactyla
Chiroptera
Pholidota
Carnivora
Rodentia
Lagomorpha
Primates
Proboscidea
Galliformes
Anseriformes
Accipitriformes
Crocodilia
Testudines
Mammalia
Aves
Reptilia

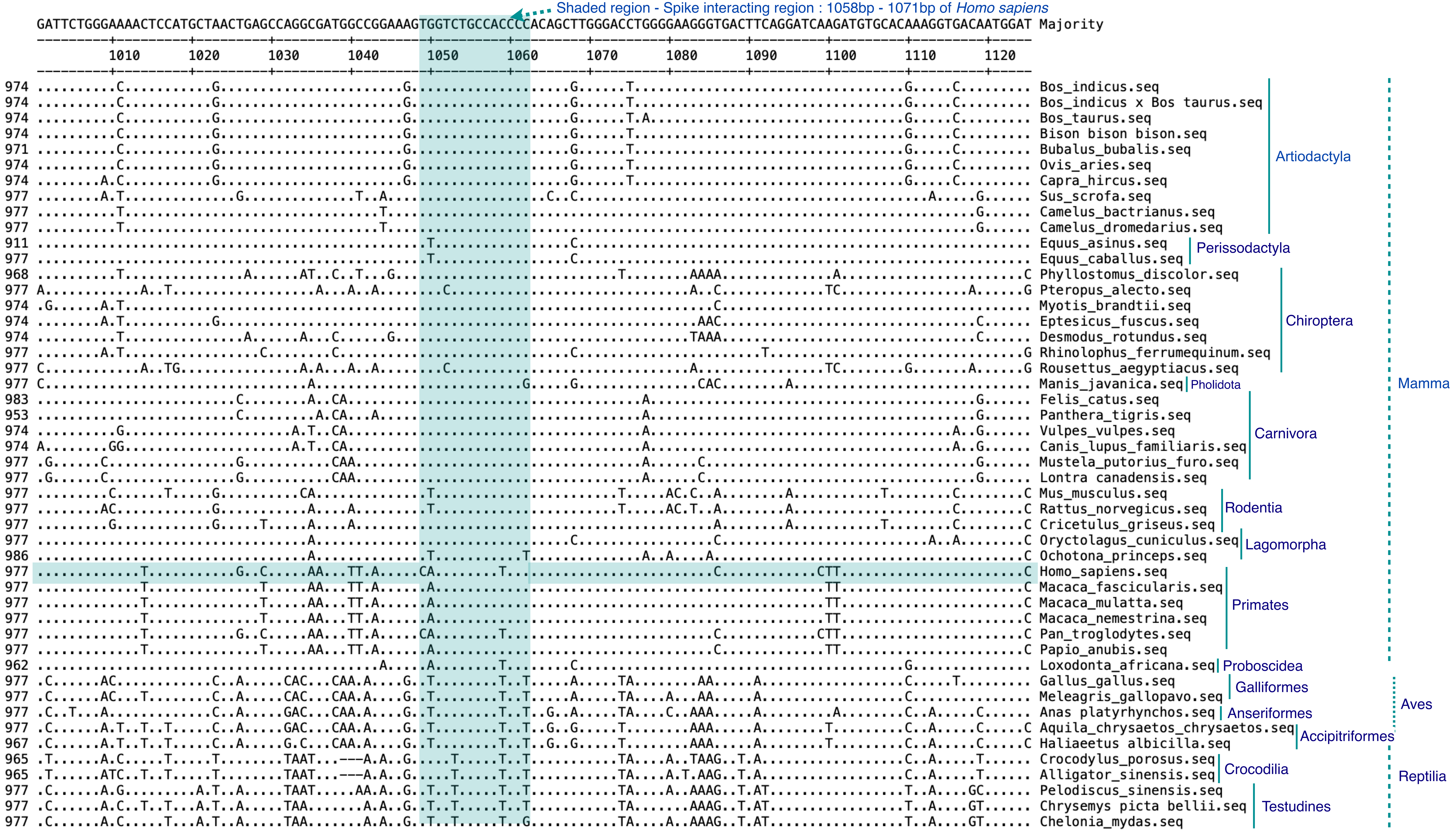

Shaded region - Spike interacting region : 1058bp - 1071bp of Homo sapiens
GATTCTGGGAAAACTCCATGCTAACTGAGCCAGGCGATGGCCGGAAAGTGGTCTGCCACCCCACAGCTTGGGACCTGGGGAAGGGTGACTTCAGGATCAAGATGTGCACAAAGGTGACAATGGAT Majority
1010 1020 1030 1040 1050 1060 1070 1080 1090 1100 1110 1120
974 Bos_indicus.seq
974 Bos_indicus x Bos taurus.seq
974 Bos_taurus.seq
974 Bison bison bison.seq
971 Bubalus_bubalis.seq
974 Ovis_aries.seq
974 Capra_hircus.seq
977 Sus_scrofa.seq
977 Camelus_bactrianus.seq
977 Camelus_dromedarius.seq
911 Equus_asinus.seq
977 Equus_caballus.seq
968 Phyllostomus_discolor.seq
977 Pteropus_alecto.seq
974 Myotis_brandtii.seq
974 Eptesicus_fuscus.seq
974 Desmodus_rotundus.seq
977 Rhinolophus_ferrumequinum.seq
977 Rousettus_aegyptiacus.seq
977 Manis_javanica.seq
983 Felis_catus.seq
953 Panthera_tigris.seq
974 Vulpes_vulpes.seq
974 Canis_lupus_familiaris.seq
977 Mustela_putorius_furo.seq
977 Lontra canadensis.seq
977 Mus_musculus.seq
977 Rattus_norvegicus.seq
977 Cricetulus_griseus.seq
977 Oryctolagus_cuniculus.seq
986 Ochotona_princeps.seq
977 Homo_sapiens.seq
977 Macaca_fascicularis.seq
977 Macaca_mulatta.seq
977 Macaca_nemestrina.seq
977 Pan_troglodytes.seq
977 Papio_anubis.seq
962 Loxodonta_africana.seq
977 Gallus_gallus.seq
977 Meleagris_gallopavo.seq
977 Anas platyrhynchos.seq
977 Aquila_chrysaetos_chrysaetos.seq
967 Haliaeetus albicilla.seq
965 Crocodylus_porosus.seq
965 Alligator_sinensis.seq
977 Pelodiscus_sinensis.seq
977 Chrysemys picta bellii.seq
977 Chelonia_mydas.seq
Artiodactyla
Perissodactyla
Chiroptera
Pholidota
Carnivora
Rodentia
Lagomorpha
Primates
Proboscidea
Mamma
Galliformes
Anseriformes
Accipitriformes
Aves
Crocodilia
Testudines
Reptilia
