# Supplementary material for: SARS-CoV-2 Spike Glycoprotein and ACE2 Interaction Reveals Modulation of Viral Entry in Wild and Domestic Animals
Source: Front Med (Lausanne). 2022 Mar 11;8:775572. doi: 10.3389/fmed.2021.775572 (PMC8962831; doi:10.3389/fmed.2021.775572)
Supplement: Data Sheet 2 — Protein sequence alignment of ACE2. The shaded regions show the spike interacting domains. [file Data_Sheet_2.PDF]

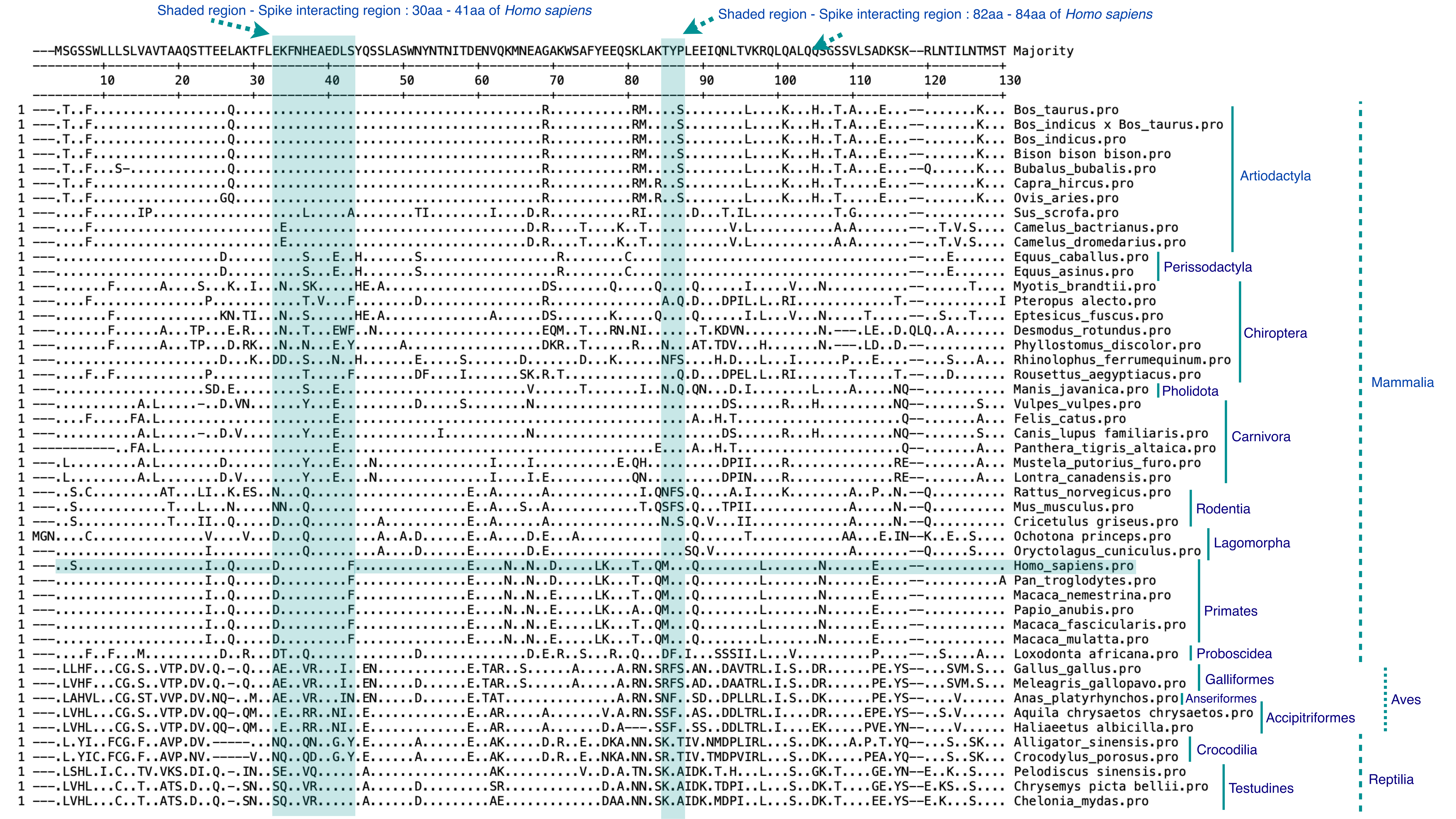
Shaded region - Spike interacting region : 30aa - 41aa of Homo sapiens
Shaded region - Spike interacting region : 82aa - 84aa of Homo sapiens
---MSGSSWLLLSLVAVTAAQSTTEELAKTFLEKFNHEAEDLSYQSSLASWNYNTNITDENVQKMNEAGAKWSAFYEEQSKLAKTYPLEEIQNLTVKRQLQALQQSGSSVLSADKSK--RLNTILNTMST Majority
10
20
30
40
50
60
70
80
90
100
110
120
130
Bos_taurus.pro
Bos_indicus x Bos_taurus.pro
Bos_indicus.pro
Bison bison bison.pro
Bubalus_bubalis.pro
Capra_hircus.pro
Ovis_aries.pro
Sus_scrofa.pro
Camelus_bactrianus.pro
Camelus_dromedarius.pro
Equus_caballus.pro
Equus_asinus.pro
Myotis_brandtii.pro
Pteropus alecto.pro
Eptesicus_fuscus.pro
Desmodus_rotundus.pro
Phyllostomus_discolor.pro
Rhinolophus_ferrumequinum.pro
Rousettus_aegyptiacus.pro
Manis_javanica.pro
Vulpes_vulpes.pro
Felis_catus.pro
Canis_lupus familiaris.pro
Panthera_tigris_altaica.pro
Mustela_putorius_furo.pro
Lontra_canadensis.pro
Rattus_norvegicus.pro
Mus_musculus.pro
Cricetulus griseus.pro
Ochotona princeps.pro
Oryctolagus_cuniculus.pro
Homo_sapiens.pro
Pan_troglodytes.pro
Macaca_nemestrina.pro
Papio_anubis.pro
Macaca_fascicularis.pro
Macaca_mulatta.pro
Loxodonta africana.pro
Gallus_gallus.pro
Meleagris_gallopavo.pro
Anas_platyrhynchos.pro
Aquila chrysaetos chrysaetos.pro
Haliaeetus albicilla.pro
Alligator_sinensis.pro
Crocodylus_porosus.pro
Pelodiscus sinensis.pro
Chrysemys picta bellii.pro
Chelonia_mydas.pro
Artiodactyla
Perissodactyla
Chiroptera
Pholidota
Carnivora
Rodentia
Lagomorpha
Primates
Proboscidea
Mammalia
Galliformes
Anseriformes
Accipitriformes
Aves
Crocodilia
Testudines
Reptilia

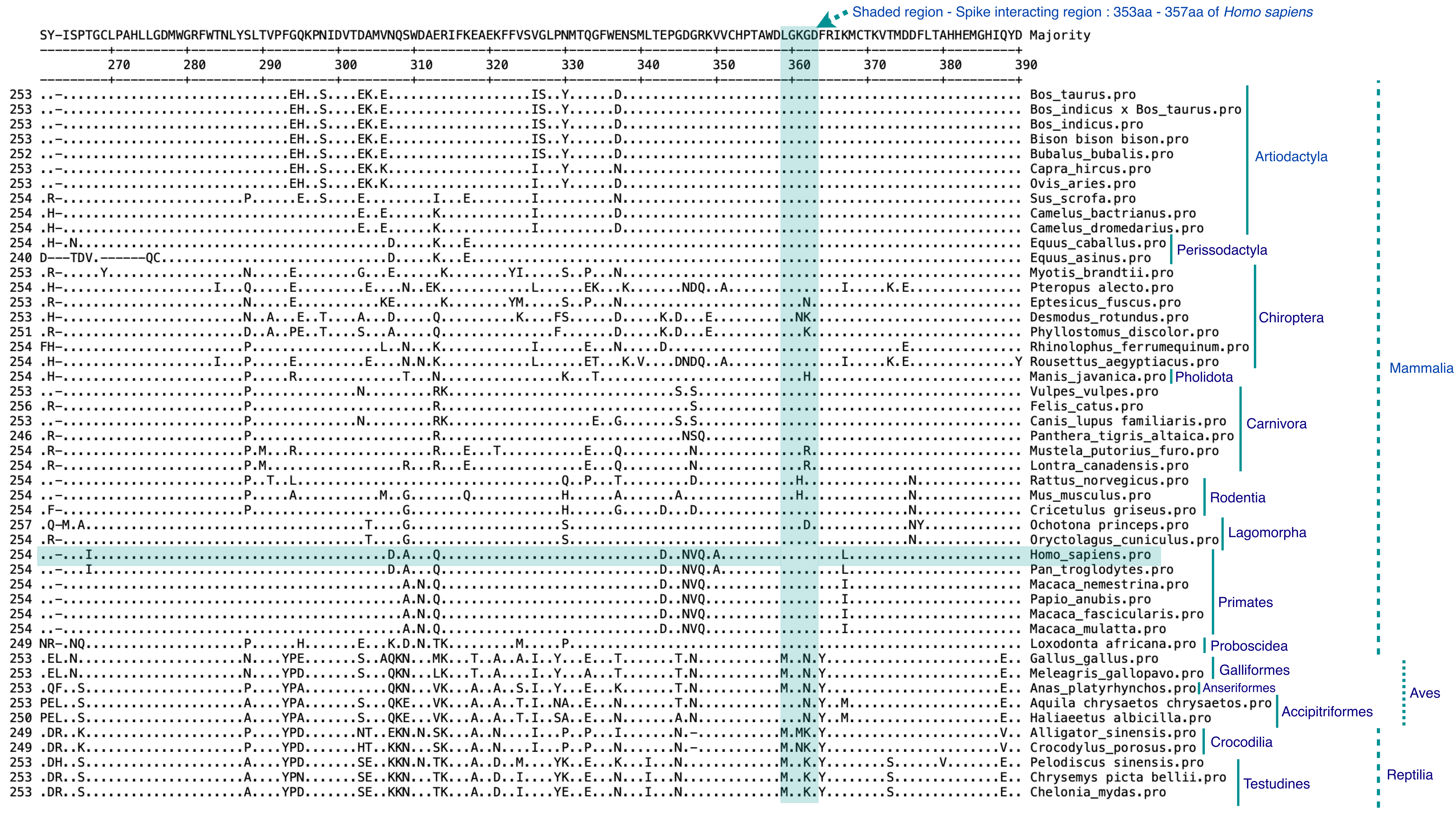
Shaded region - Spike interacting region : 353aa - 357aa of Homo sapiens
SY-ISPTGCLPAHLLGDMWGRFWTNLYSLTVPFGQKPNIDVTDAMVNQSWDAERIFKEAEKFFVSVGLPNMTQGFWENSMLTEPGDGRKVVCHPTAWDLGKGDFRIKMCTKVTMDDFLTAHHEMGHIQYD Majority
270 280 290 300 310 320 330 340 350 360 370 380 390
Bos_taurus.pro
Bos_indicus x Bos_taurus.pro
Bos_indicus.pro
Bison bison bison.pro
Bubalus_bubalis.pro
Capra_hircus.pro
Ovis_aries.pro
Sus_scrofa.pro
Camelus_bactrianus.pro
Camelus_dromedarius.pro
Artiodactyla
Equus_caballus.pro
Equus_asinus.pro
Perissodactyla
Myotis_brandtii.pro
Pteropus alecto.pro
Eptesicus_fuscus.pro
Desmodus_rotundus.pro
Phyllostomus_discolor.pro
Rhinolophus_ferrumequinum.pro
Rousettus_aegyptiacus.pro
Chiroptera
Manis_javanica.pro
Pholidota
Vulpes_vulpes.pro
Felis_catus.pro
Canis_lupus familiaris.pro
Panthera_tigris_altaica.pro
Mustela_putorius_furo.pro
Lontra_canadensis.pro
Carnivora
Rattus_norvegicus.pro
Mus_musculus.pro
Cricetulus griseus.pro
Rodentia
Ochotona princeps.pro
Oryctolagus_cuniculus.pro
Lagomorpha
Homo_sapiens.pro
Pan_troglodytes.pro
Macaca_nemestrina.pro
Papio_anubis.pro
Macaca_fascicularis.pro
Macaca_mulatta.pro
Primates
Loxodonta africana.pro
Proboscidea
Mammalia
Gallus_gallus.pro
Meleagris_gallopavo.pro
Galliformes
Anas_platyrhynchos.pro
Anseriformes
Aquila chrysaetos chrysaetos.pro
Haliaeetus albicilla.pro
Accipitriformes
Aves
Alligator_sinensis.pro
Crocodylus_porosus.pro
Crocodilia
Pelodiscus sinensis.pro
Chrysemys picta bellii.pro
Chelonia_mydas.pro
Testudines
Reptilia
