# Supplementary figures and images for: SARS-CoV-2 Spike Glycoprotein and ACE2 Interaction Reveals Modulation of Viral Entry in Wild and Domestic Animals
Source: Front Med (Lausanne). 2022 Mar 11;8:775572. doi: 10.3389/fmed.2021.775572 (PMC8962831; doi:10.3389/fmed.2021.775572)

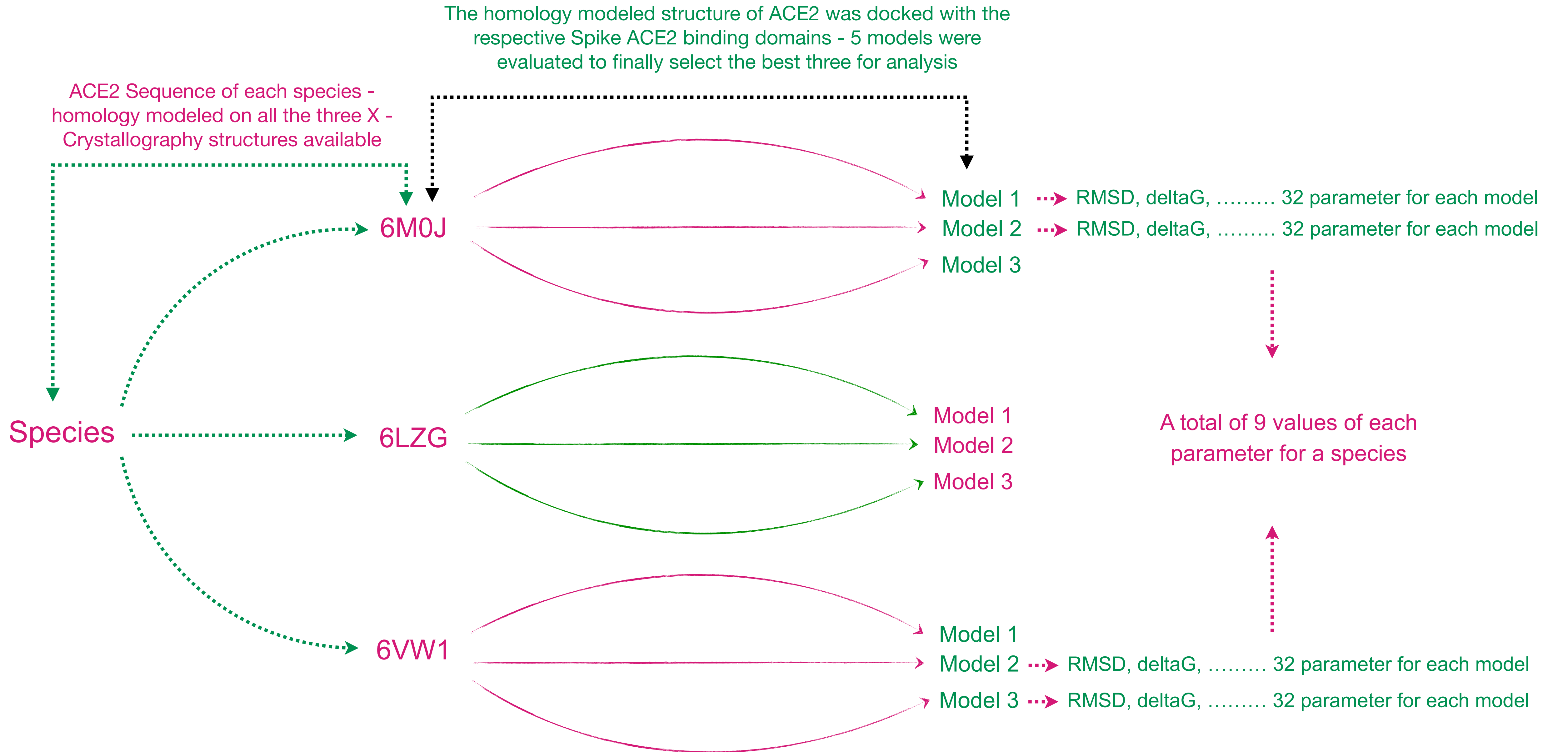

Supplement: Data Sheet 3 — Depiction of numbers of models considered in this study showing the number of values per parameter. For each species, the ACE2 sequence is homology modeled against the three X-crystallography structures— 6M0J, 6LZG, and 6VW1. The spike ACE2-binding domain of each of the X-crystallography structures is docked with its homology modeled ACE2 and 5 docked complexes were evaluated to select the top three models. This leaves us with 9 values for all the spike binding parameters for further analysis. [file Data_Sheet_3.PDF]
