# Supplementary material for: SARS-CoV-2 Spike Glycoprotein and ACE2 Interaction Reveals Modulation of Viral Entry in Wild and Domestic Animals
Source: Front Med (Lausanne). 2022 Mar 11;8:775572. doi: 10.3389/fmed.2021.775572 (PMC8962831; doi:10.3389/fmed.2021.775572)
Supplement: Supplementary Table 8 — List of significant spike binding parameters after unpaired t-test between the known infected and uninfected groups. [file Table_8.DOCX]

**Parameters that are significant between the infected and the uninfected on Unpaired t-test**

| Parameter | Significance (** - 1% level of significance and * - 5% level of significance) |
| --- | --- |
| RMSD | S** |
| deltaG | S* |
| Van_der_Waals | S* |
| entropy_sidechain | S* |
| Solvation_Hydrophobic | S** |
| IntraclashesGroup1 | S** |
